# Fatal fulminant herpes simplex hepatitis secondary to tongue piercing in an immunocompetent adult: a case report

**DOI:** 10.1186/1752-1947-2-356

**Published:** 2008-11-20

**Authors:** Shaheen E Lakhan, Lindsey Harle

**Affiliations:** 1 Global Neuroscience Initiative Foundation, Los Angeles, CA, USA

## Abstract

**Introduction:**

Herpes simplex infection is most commonly a benign, self-limiting disease with mucocutaneous lesions and mild viremia. Immunosuppressed patients are at a higher risk of disseminated infection, as are neonates and pregnant women. The incidence of fulminant herpes simplex virus hepatitis is extremely low, and the diagnosis is often missed due to the lack of specific signs or symptoms.

**Case presentation:**

We present the case of an immunocompetent, previously healthy young woman who contracted herpes simplex virus, presumably through a recent tongue piercing, which progressed to fulminant hepatitis and death.

**Conclusion:**

Despite aggressive medical therapy, fulminant herpes simplex virus hepatitis is fatal in the majority of patients. We present a review of the literature, which shows that immunocompetent adults have rarely been affected by fulminant herpes simplex virus hepatitis. Initiation of empirical therapy is warranted in patients with progressive hepatic failure with no other underlying cause. Acyclovir therapy has proven effective in some patients, but is less effective in patients who present in advanced stages of infection.

## Introduction

Herpes simplex infection is very common and affects all ages. Most commonly, it presents as a benign, self-limiting disease with mucocutaneous lesions and mild viremia. Individuals who are immunocompromised, neonates, and pregnant women are at a higher risk of widespread disseminated infection including hepatitis. The incidence of fulminant HSV hepatitis is extremely low, and the diagnosis is often missed due to the lack of specific signs or symptoms.

## Case presentation

The patient was a 19-year-old Caucasian woman who presented to the clinic initially with nonspecific symptoms of fatigue, fever and abdominal pain. Past medical history was noncontributory; she was an otherwise healthy adult from the United States, and did not report ill contacts, intravenous drug use, or recent sexual contacts. She had a temperature of 102.3°F, WBC of 4,800, AST of 330 U/liter and ALT of 250 U/liter. She was thought at the time to have a viral prodrome and was treated symptomatically. The patient returned to the clinic 3 days later with resolution of her constitutional symptoms but with the development of inflammation and pain around her recent tongue piercing (1 to 2 weeks before this visit). The patient was treated for oral thrush, and cultures of the tongue were taken and grew normal oral flora and beta hemolytic streptococci group C. Several days later, the patient presented to the emergency room with worsening fever, abdominal pain, vomiting, diarrhea, myalgia, and arthralgia. At this time, she had an AST of 6000 U/liter and ALT of 4000 U/liter. The following day, her lab values increased to an AST of 9200 U/liter and an ALT of 4400 U/liter. Bilirubin and alkaline phosphatase were within normal limits. Other laboratory values were as follows: alcohol, non-detectable; CMV, infectious mononucleosis, leptospira, EBV, HBV, HCV, HEV, HIV all negative; urine drug screen negative; serum acetaminophen level of 3 μg/dl.

CT scan showed a mottled liver and a 2 to 4 mm abscess of the anterior tongue. Shortly after admission to the ICU, she developed hypotension, coagulopathy with a PT of 83.2s and PTT of greater than 200s, hyperammonemia, and acute renal failure thought to be due to hepatorenal syndrome. The medical team was in the process of preparing her for transport to receive a liver transplant but the patient expired. Postmortem laboratory results revealed a tongue viral culture positive for HSV and a positive serum HSV PCR.

**Figure 1:**
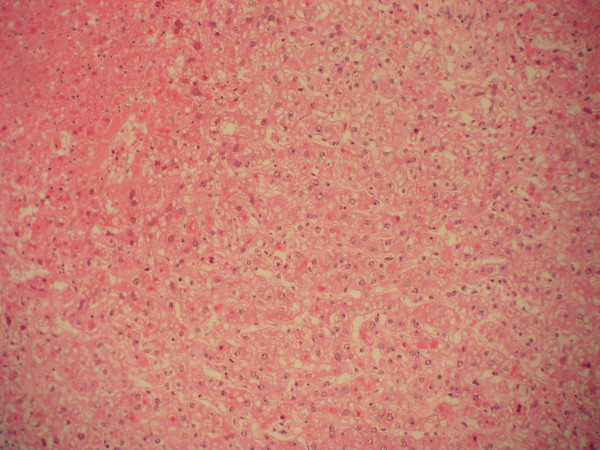
Liver, medium power, demonstrating diffuse necrosis and loss of normal architecture.

**Figure 2:**
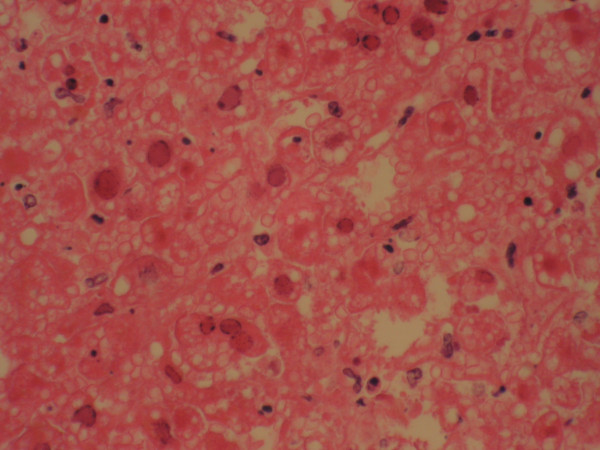
Liver, high power, demonstrating Cowdry bodies within hepatocytes.

**Figure 3:**
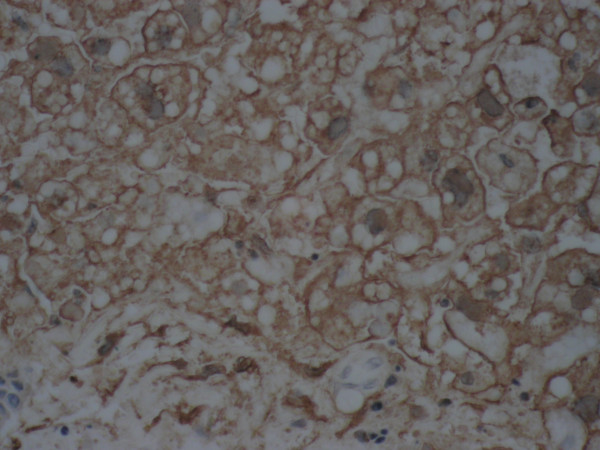
Liver, high power, immunohistochemical staining for HSV-1, demonstrating diffuse positivity.

Autopsy revealed a liver weighing 1620 g with diffuse geographic necrosis. Histological examination of the liver showed extensive hemorrhagic necrosis with HSV intranuclear inclusion (Figure 1), Cowdry type 1 and 2 (Figure 2), with immunoreactivity for HSV-1 (Figure 3). Other findings included bilateral pleural effusions (approximately 500 ml) and a pelvic wall hematoma (4.0 × 2.5 cm).

## Discussion

Herpes simplex virus (HSV) hepatitis occurs most commonly in the setting of immunocompromise, but has also been reported in immunocompetent adults, children, and pregnant women [1-3]. To our knowledge, only seven cases of fulminant hepatitis due to HSV in immunocompetent adults have been reported. However, it is likely that many cases go undetected due to the nonspecific clinical and laboratory presentation. Patients with HSV hepatitis can present with a wide range of symptomatology, from mild constitutional symptoms to severe coagulopathy with loss of consciousness [4]. Early diagnosis of HSV hepatitis is imperative in order to institute treatment in a timely manner. The mortality rate is high among untreated patients [5]. In one review, only 23% of reported patients were diagnosed antemortem [6]. Untreated herpes hepatitis is associated with mortality in 80% to 90% of cases [7].

Early initiation of antiviral therapy, especially acyclovir, can improve chances of survival [8,9].

There is no diagnostic pattern to the presentation of HSV hepatitis. Patients present with symptoms such as fever and abdominal pain in combination with rising ALT and AST titers [3,10]. In a review of 137 cases of HSV hepatitis, the most common presenting features were fever (98%), coagulopathy (84%), and encephalopathy (80%). Rash was seen in less than half of patients [11]. Over half of cases (58%) were first diagnosed at autopsy, and three-quarters of the cases (74%) progressed to death or liver transplantation. Other abnormalities that may be present in patients with HSV hepatitis include leukopenia, serological evidence of infection, and mucocutaneous lesions, but these factors are not present in all patients.

Fulminant HSV hepatitis is usually marked by significant elevations in transaminases, with AST typically higher than ALT, and a mild or absent hyperbilirubinemia. Serological testing for HSV-IgM and -IgG is often negative, however, it does not rule out HSV as the underlying etiology.

Definitive diagnosis is made by liver biopsy, with demonstration of hepatic necrosis, HSV cytopathic effects, and immunoreactivity to HSV [3]. Viral blood cultures will not provide timely results, and real-time PCR testing for viremia, which can provide results in 3 hours [12], is not available at every center.

Levels of ALT and AST correlate with survival. A greater than 100-fold increase in ALT and AST was associated with fatality in 100% of patients in one review [12]. Liver biopsy and blood cultures should be performed, before initiation of antiviral therapy, but empiric therapy should be instituted immediately in patients with no other known reason for hepatic failure.

Biopsy will demonstrate diffuse hepatic necrosis with hemorrhage, and may demonstrate Cowdry type 1 and 2 inclusions. Collapse of the normal architecture with loss of the reticulin framework will be present. A lymphocytic infiltration may be seen, but is usually modest. Immunostaining for HSV will detect the presence of the virus within the hepatocyte cytoplasm. Blood cultures provide supportive evidence to the diagnosis.

A high index of suspicion is necessary when a patient presents with constitutional symptoms and elevating AST and ALT, and these findings should prompt immediate antiviral therapy with acyclovir. Risk factors for HSV hepatitis include third trimester pregnancy and immunosuppression. The degree of elevation of AST and ALT should be taken into account, because many viral illnesses, including primary HSV infection, can produce mild elevations in liver enzymes without progression to fulminant hepatitis. Serial evaluation of these values will show consistent increase when hepatic damage is severe, and should prompt immediate intervention. Even in the absence of direct evidence of acute HSV infection, administration of acyclovir is a relatively safe treatment. While fulminant hepatitis is only rarely due to HSV, the fact that this infection often responds to antivirals early in its course warrants empirical treatment. Despite this, there has been a report of acyclovir-resistant HSV hepatitis [13].

Body piercing is a known risk factor for HSV infection. A review by Hayes and Harkness reported HSV infection and/or seroconversion to be associated with percutaneous needle exposure and body piercing [14]. These findings indicate the need for public health intervention, including education and regulation of body piercing practices, in order to prevent transmission of HSV.

## Conclusion

Clinicians should have a high index of suspicion for HSV hepatitis in both immunocompetent and immunocompromised patients with elevated liver enzymes and no other underlying disease. Fulminate hepatitis may occur without evidence of primary HSV infection. Acyclovir treatment should be initiated early in cases of hepatitis of unknown etiology, as early initiation of therapy is imperative to prevent severe disease resulting in liver transplantation or death.

## Abbreviations

ALT: alanine transaminase; AST: aspartate aminotransferase; CMV: cytomegalovirus; CT: computed tomography; EBV: Epstein-Barr virus; HBV: hepatitis B virus; HCV: hepatitis C virus; HEV: hepatitis E virus; HIV: human immunodeficiency virus; HSV: herpes simplex virus; ICU: intensive care unit; PCR: polymerase chain reaction; PT: prothrombin time; PTT: partial thromboplastin time; WBC: white blood cell count

## Consent

Written informed consent was obtained from the next-of-kin of the patient for publication of this case report and any accompanying images. A copy of the written consent is available for review by the Editor-in-Chief of this journal.

## Competing interests

The authors declare that they have no competing interests.

## Authors' contributions

SL and LH secured the case, conducted the literature review, and participated in the preparation of the manuscript. Both authors read and approved the final manuscript.
